# Supplementary material for: Effects of LED Light Colors on the Growth Performance, Intestinal Morphology, Cecal Short-Chain Fatty Acid Concentrations and Microbiota in Broilers
Source: Animals (Basel). 2023 Dec 1;13(23):3731. doi: 10.3390/ani13233731 (PMC10705592; doi:10.3390/ani13233731)
Supplement: Supplementary file 1 [file animals-13-03731-s001.zip › animals-2690885-supplementary.pdf]

Supplementary Materials

Supplementary Table S1. Composition and nutrient levels of diets.

Items	1 to 21 d	22 to 42 d
Ingredients (g/kg)		
Corn	42.10	42.30
Wheat	20.00	25.00
Soybean meal	28.40	21.20
Corn gluten meal	4.00	4.50
Soybean oil	1.50	3.00
Premix [1]	4.00	4.00
Total	100.00	100.00
Nutrient levels [2] (g/kg)		
ME[2,4]/(MJ/kg)	12.30	12.97
CP[3,4]	20.92	20.35
Calcium[4]	1.00	1.00
Available phosphorus[4]	0.40	0.45
Lysine[4]	1.15	1.10
Methionine[4]	0.52	0.41

[1] The premix provided the following per kg of the diets: Vitamin A 120000 IU，Vitamin D_3 2500 IU，Vitamin E 20 mg，Vitamin K_3 3 mg，Vitamin B_1 3 mg，Vitamin B_2 8 mg，Vitamin B_{12} 0.03 mg，Pantothenic 20 mg，Nicotinic acid 50 mg，Biotin 0.1 mg，Folic acid 1.5 mg，Cu 9 mg，Zn 110 mg，Fe 100 mg，Mn 100 mg，Se 0.16 mg，I 0.6 mg;

[2] ME: Metabolizable energy;

[3] CP: Crude protein;

[4] All values except the amino acids and available phosphorus are measured.

Supplementary Table S2. The average body weight of the groups

Periods	Average body weight (g)			
	White light	Green light	Blue light	Blue-green light
21 d	797.22	780.34	826.93	796.19
42 d	2341.28	2211.00	2485.38	2449.17

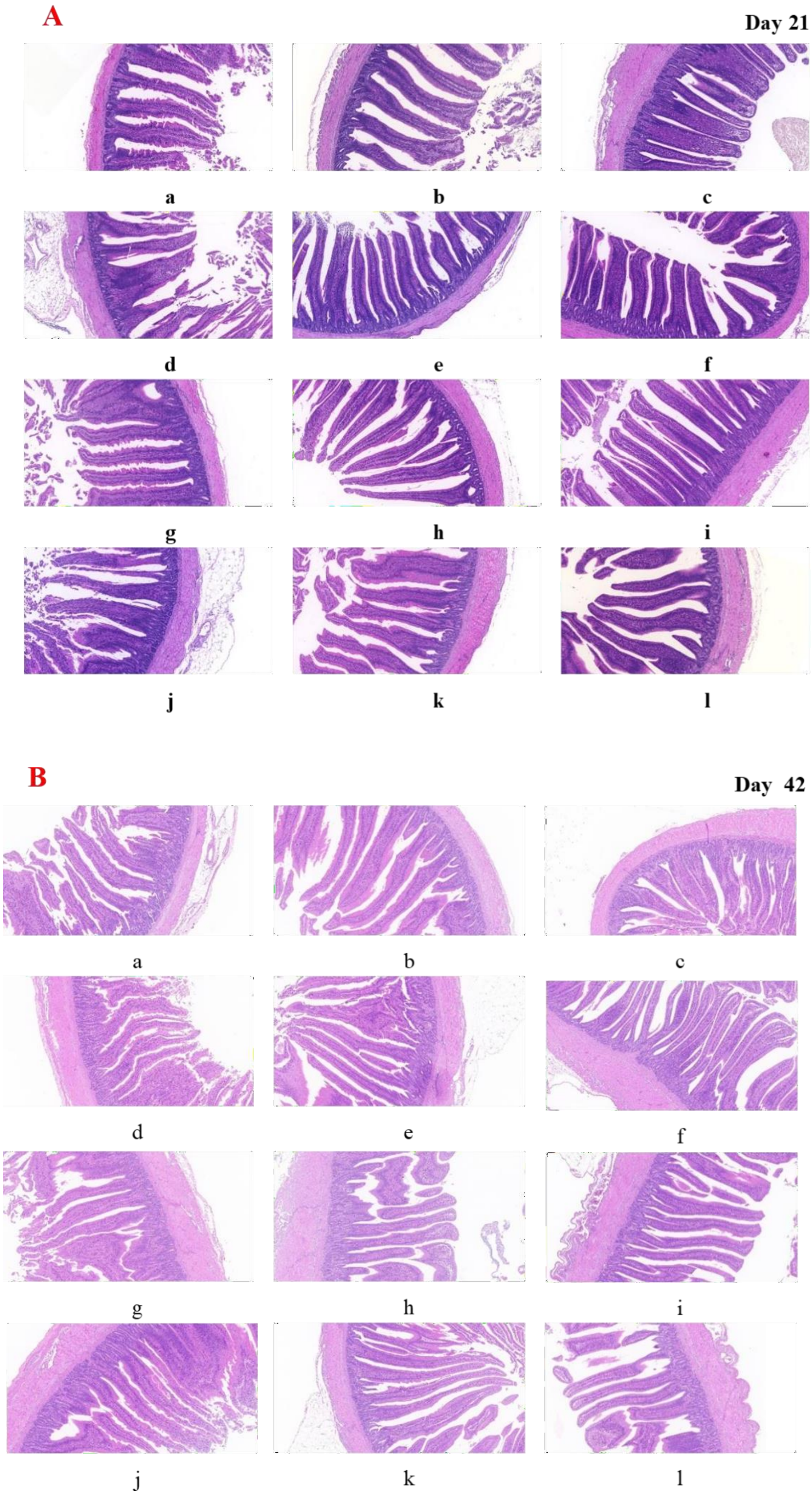

Supplementary Figure S1. Morphology of intestinal mucosa in each group at day 21(A) and at day 42 (B); (a) duodenum of W group; (b) jejunum of W group; (c) ileum of W group; (d) duodenum of G group; (e) jejunum of G group; (f) ileum of G group; (g) duodenum of B group; (h) jejunum of B group; (i) ileum of B group; (j) duodenum of BG group; (k) jejunum of BG group; (l) ileum of BG group.

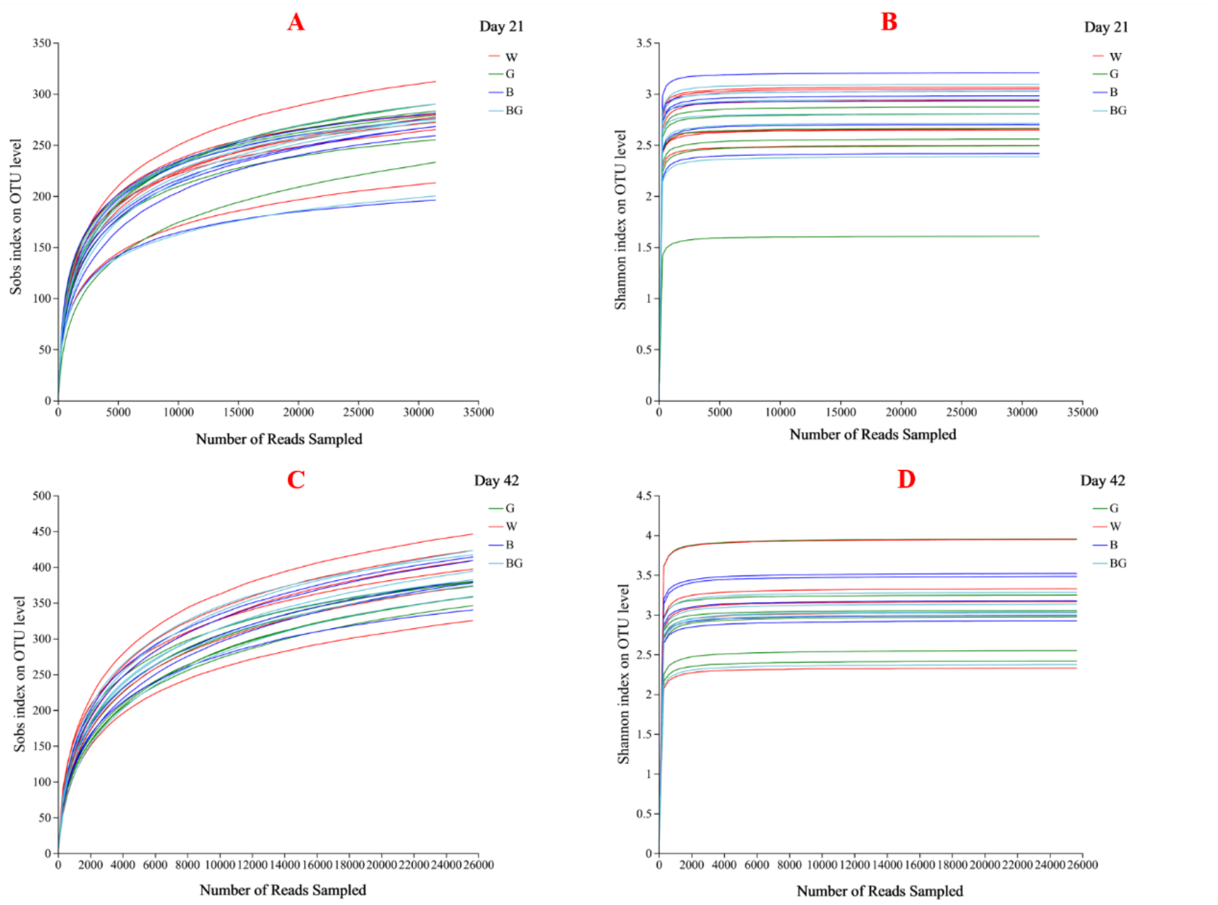

Supplementary Figure S2. Rarefaction curve; (A) Sobs rarefaction curve on day 21; (B)Shannon rarefaction curve on day 21; (C) Sobs rarefaction curve on day 42; (D) Shannon rarefaction curve on day 42.
